# Would Organizational Climate and Job Stress Affect Wellness? An Empirical Study on the Hospitality Industry in Taiwan during COVID-19

**DOI:** 10.3390/ijerph181910491

**Published:** 2021-10-06

**Authors:** Pei-Ling Tsui

**Affiliations:** 1 Department of Hospitality Management, National Taitung Junior College, Taitung 95045, Taiwan; tsui68@ntc.edu.tw; 2 Graduate Institute of Technological and Vocational Education, National Taipei University of Technology, Taipei 10608, Taiwan

**Keywords:** COVID-19, organizational climate, environmental job stress, wellness, hospitality industry

## Abstract

During the COVID-19 pandemic, hospitality employees face a tremendous amount of job stress due to the decline in revenue and close contact with people. This study has three aims: first, to analyse the status quo of organizational-climate job stress on employee wellness in the hospitality industry during COVID-19; second, to discuss the correlation between organizational-climate job stress and employee wellness in the hospitality industry; and third, to analyze the associations between of personal background and organizational climate on job stress and wellness in the hospitality industry. This research uses a survey method to examine these issues. Participants were employees of franchise hotel branches in Taipei City, which yielded 295 effective sample sizes from five chain hotels. The personal background factor questionnaire, organizational climate questionnaire, job stress questionnaire, and wellness questionnaire served as the main research tools. In this study, Factor analysis, Pearson Correlation and Multiple Regression Analysis were used for sample analysis. The results revealed a significant relationship between organizational-climate job stress with wellness. Personal background factors, organizational climate, and job stress would affect the wellness of employees. As a result, the present research provides empirical evidence for the impact of organizational climate and job stress on employee wellness in the hospitality industry in Taiwan during COVID-19. The study’s findings, as well as its theoretical and practical implications, are discussed. The main contribution of this study is that the results serve as a reference for hospitality business owners to design better organizational environments for their employees, plan human-resource-related strategies, and provide training for their employees during a pandemic.

## 1. Introduction

A working environment that attends more closely to the needs of employees would increase their willingness to spend more time and effort on their work tasks [1,2,3]. Organizational climate is an important concept that drives the aforementioned scenario. Organizational climate, an enduring quality of the internal environment of an organization, exerts impacts on the behaviours of organizational members [4]. Marinova and Park [5] found that organizational climate could predict employees’ work attitudes and cognition, as well as allowing managers to make preparations and improve the workplace. Job stress refers to an individual’s perceived feeling that they need to deviate from normal expectations when they have to handle important work-related opportunities, restrictions, or requirements. An individual may perceive an inappropriate level of stress or burden when their own adjustment capacity is constantly in conflict with the events that surround them [6,7].

In recent years, the hospitality industry in Taiwan has suffered a great decline in revenue due to the impact of COVID-19. Moreover, Taiwan began to restrict the entry of foreign nationals in late March 2020 and completely banned indoor dining in early May 2021 due to the alarming epidemic. These measures drastically altered consumer behaviours and resulted in the sharp decline of revenue from catering and tour operators, as well as the negative growth of the hospitality industry in Taiwan [8]. Faced with unprecedented business challenges, hospitality workers constantly have job stress, and business owners even required them to achieve a decent business performance in order to overcome the COVID-19 crisis. As a result, job stress became concomitant with the pandemic [9,10,11].

According to the World Health Organization (WHO) in 1946, health is a state of complete physical, mental, and social wellness, and not merely the absence of disease or infirmity. This definition implies that health should cover physical, mental, and social aspects. Physiological diseases and psychological impacts may arise when individuals are unable to alleviate the tremendous level of stress that they perceive [12,13,14]. To prevent the negative impact of stress, the pursuit of wellness is seemingly the ultimate goal of hospitality employees.

The hospitality industry is essential in a modern country, as well as an important indicator that reflects the level of the country’s development and the quality of life of its citizens [15]. Globalization developments during the COVID-19 pandemic have intensified the competition within the hospitality industry. Therefore, the approaches employed by Taiwanese hospitality business owners in response to the course of the epidemic are remarkably important, particularly because these approaches must be able to strengthen organizational structure, reduce job stress, and enhance the well-being of employees. On this basis, this research conducted further research and analysis on the impact of organizational climate and job stress on the wellness of Taiwanese hospitality employees.

According to the aforementioned discourse, the objectives of this study are as follows:

I. To analyse the current status of organizational climate, job stress and wellness in Taiwan’s hospitality industry during the COVID-19 pandemic.
II. To explore the correlations between organizational climate, job stress, and wellness in the hospitality industry.
III. To analyze the associations between personal background and organizational climate on job stress and wellness in the hospitality industry.

## 2. Materials and Methods

### 2.1. Organisational Climate

Organizational climate is a general concept constituted by employees’ subjective opinions about their organization, management, and other environmental factors. It represents a group of attributes that are used to describe an organization’s behaviour. While the organizational climate of some organizations is open and harmonious, some instead are dour and depressing. These different organizational climates exert different impacts on the behavioural intentions and work ethic of members. Relevant studies also show that employee fringe benefits and a supportive work climate correlate positively with job satisfaction and enthusiasm, and also reduce job stress among employees [16,17]. Employee opinions toward the workplace may be representative of the organizational climate [18]. Shanker et al. [19] pointed out in his study that innovative organizational behaviours have emerged as a material topic, as innovative organizational climate has an indivisible impact on organizational performance. Ahmad et al. [20] maintained that organizational climate is among the factors that would impact an individual’s understanding of their capabilities. In the service industry, organizational climate is perceived to be more important than work tasks [17,21]. Taken together, Litwin and Stringer’s [22] theory serves as the theoretical basis of this study.

### 2.2. Job Stress

As defined by the American National Institute for Occupational Safety and Health in 1999, job stress is the harmful physical and emotional responses that occur when the requirements of a job do not meet the capabilities, resources, or needs of the worker. There are many factors and sources that could lead to job stress, such as interpersonal relationships, organizational management approaches, work overload, long working hours, or repetitive work tasks, etc. which could gravitate towards job stress [23]. Hospitality employees often perceive more stress as they need to strike a balance between their job and family responsibilities [15,24,25].

Stress is neither good nor bad. Although the negative effects of stress are often visible, stress has positive effects, especially when it creates opportunities in potential fields of interest [26]. The perception of job stress is closely related to the traits of an individual. If an individual responds appropriately to stress, they would be able to mitigate the negative impacts of job stress on their life, mind, and body. Excessive stress, on the other hand, drags the individual down, which not only reduces their work efficiency and influences their interpersonal relationships but also decreases their wellness [27]. Koc and Bozkurt [28] pointed out in their study that job stress comes from a variety of complex sources, among which are mainly related to the external environment, jobs, and the individuals themselves. These factors are also influenced by various intricate and subtle factors that are subjective and objective. Based on the aforementioned elaborations, this study identifies the components of job stress as workload, job support stress, interpersonal stress, and professional knowledge stress.

### 2.3. The Hospitality Industry and Job Stress

In the hospitality industry, employees are required to serve customers in a kind and gracious manner. When employees fail to meet their own inner requirements, they must suppress their negativity and continue to present themselves externally as enthusiastic and dedicated. This emotional burden may result in burnout and fatigue [29]. Job stress is usually manifested as lethargy. Employees would feel exhausted and tired after work and slack off before starting work. Therefore, the sense of burnout at work weakens employees’ motivation to pursue and achieve good performances, which in turn leads to physical and mental loss. Stress gives the feeling that one finds it difficult to complete their work tasks [15,30].

In the fiercely competitive hospitality industry, considerable importance is attached to service quality and the provision of high-quality products. In light of growing consumer expectations, workers must keep an enthusiastic and friendly attitude and take positive actions [31]. Indeed, hospitality employees often experience tremendous levels of job stress [32]. Comprehensive stress-related factors include perception of burnout and mental factors such as anxiety, frustration, hostility, and insecurity. The degree of impact of these factors depends on the intensity of stress and stressors [33].

These stressors are related to working conditions (shift work, extreme kitchen temperature), the roles and tasks performed (work overload, nonstandard work arrangements), as well as social and emotional job climate, such as workplace bullying and emotional dissonance [34]. These stressors influence an individual’s physical (such as illness, symptoms, etc.) and mental (such as emotions) health due to temporal and expectation-related conflicts. Since service quality in the hospitality industry is correlated with customer loyalty and brand image, absence from duty without leave becomes more prevalent due to job stress, work-family conflicts, customer service, work shifts, and the lack of employment security. In particular, employees may sacrifice their physical and mental health for the sake of good service quality, and employees who are often in direct contact with customers are at a higher risk of spreading viral diseases [11,35].

The hospitality industry requires work shifts, which may increase the risk of short-term health impacts including work-related injuries and accidents. Additionally, prolonged work shifts may also affect the cardiovascular system, metabolism, digestive system, immune system, and hormone balance. Occupational injuries, such as mental disease, physical injury, medical malpractices, job stress, etc., would also lower job satisfaction [15,36]. However, providing excellent services is a prerequisite for hospitality employees, which means that their service value must be reflected through their professional competence and ability to provide high-quality services. Therefore, the operations and developments of businesses in the hospitality industry rely on the professionalism, dedication, and meticulousness of employees. This study explores the associated of organizational climate and job stress on the physical and mental state of hospitality employees, with the expectation that more attention can be drawn to the work environments of hospitality employees and further precautions can be taken.

### 2.4. Wellness

Eisenberg [37] and Engel [38] argued that disease is a negative subjective experience while wellness is a positive subjective experience. In terms of health and perception, individuals can be healthy but feel uncomfortable, or feel good but are unhealthy. Therefore, the WHO has taken into account an individual’s self-reported health status as the well-being perceived by staying healthy and taking more positive actions [39]. Moreover, good health, considered to be pain-free in the past, has been gradually re-interpreted by the concept of wellness. Additionally, the concept of wellness has received considerable attention and is interpreted as health, comfort, total fitness, holistic health, and happiness, etc., as well as covering physical, physiological, emotional, social, environmental, and occupational aspects.

Wellness is considered as an individual’s life satisfaction or positive state of mind. The definition of health is also impacted by positive psychology. The criteria for measuring wellness include dedication, interpersonal relationships, significance of existence, and sense of personal achievement. On the positive level, wellness is a robust feeling of physical and mental health [40].

Russell [41] argued that health is an individual’s state of complete physical, social, and physiological comfort, rather than merely being free from illness. The evaluation of personal wellness generally covers physical and mental aspects. In terms of physical health, health is the self-reported feeling of physical or physiological functioning. Objective evaluations are performed through diagnoses of chronic and acute diseases as well as declines and restrictions in physical functioning; while subjective evaluations are performed through self-reported perceptions and attitudes [42]. With regard to physiological health, individuals subjectively examine whether they are in a good mental state and have good social adaptability through feelings of anxiety, worry, interpersonal adaptation, depression, emotional anxiety, emotional tension, and life adjustment [43]. More specifically, healthiness not only refers to being in good health and free from illnesses, but also feeling comfortable in all aspects, including the physical, mental, and social aspects [44]. Therefore, besides the basic needs to survive, health also entails the pursuit of the value of life and wellness [45,46]. Based on Russell’s [41] arguments, this research defines wellness as an individual’s mental health and physical health.

According to the above literature, this research proposes the following hypotheses:

**Hypothesis** **1.** *Organizational climate and job stress are associated with wellness.*

**Hypothesis** **2.** *Personal background and organizational climate associated job stress and wellness.*

## 3. Materials and Methods

### 3.1. Study Design and Procedure

The present study follows a cross-sectional survey design, using questionnaires for data collection. The researchers contacted supervisors in five franchise hotels in Taipei City, Taiwan by e-mail. They explained the purpose and objectives of the research, and the supervisors agreed to distribute a questionnaire to the hotel’s employees.

### 3.2. Ethical Statement

Because no therapeutic medication was involved in this study, it required no formal approval from the Institutional Review Board of the local Ethics Committee. Nonetheless, all respondents were informed about the study’s purpose and participation was voluntary. Participants were assured of the confidentiality and anonymity of all information associated with the surveys. The study was conducted according to the Declaration of Helsinki guidelines.

### 3.3. Sampling, and Recruitment

Using snowball sampling, this study recruited employees from five chain hotels in Taipei City. The researchers issued 330 questionnaires from January to June 2021, from which they received 295 valid completed questionnaires. Participants were recruited after the researchers posted explanatory posters in staff restaurants and provided a mailbox for the completed questionnaires. Participants filled out the anonymous questionnaire, and confidentiality was respected.

### 3.4. Measuring Tools

In this empirical study, a questionnaire was devised in this study to collect information about the associations between of organizational climate and job stress on wellness. This research applied four measuring tools, namely (1) a personal background questionnaire; (2) an organizational climate questionnaire; (3) a job content questionnaire; and (4) a well-being questionnaire. The scales in this research were revised by three hospitality management college professors and five hotel supervisors in human resource departments. The phrasing of the questionnaires complied with the titles of organizational climate, job stress, and wellness of hospitality employees during COVID-19 in Taiwan.

#### 3.4.1. Organizational Climate Questionnaire

This research edited and revised the organizational climate questionnaire developed by Litwin and Stringer [22]. The questionnaire covers three dimensions, namely Reward and Promotion Incentive, Identification and Responsibility. Scores are measured on a five-point Likert scale, with the measures being strongly agree (5 points), agree (4 points), neutral (3 points), disagree (2 points), and strongly disagree (1 point). The higher the score, the stronger the participant’s positive perception of the organizational climate, and vice versa. The internal consistency of the scale expressed as the Cronbach’s alpha is 0.896, suggesting that the questionnaire has good reliability. This research uses the Kaiser-Meyer-Olkin (KMO) test to sample goodness of fit, Bartlett’s sphericity test, and exploratory factor analysis to verify its construct validity (see Table 1).

#### 3.4.2. Job Content Questionnaire (JCQ)

This study edited and revised the job content questionnaire developed by Karasek et al. [47]. The scale consists of four dimensions—namely Workload Stress, Interpersonal Stress, Supervisor Support Stress, and Professional Competence Stress. Scores are measured on a five-point Likert scale, with the measures being strongly agree (5 points), agree (4 points), neutral (3 points), disagree (2 points), and strongly disagree (1 point). The higher the score, the stronger the participants’ perception of job stress, and vice versa. The internal consistency of the scale expressed as the Cronbach’s alpha is 0.859, suggesting that the questionnaire has good reliability. This research uses the KMO test for goodness of fit, Bartlett’s sphericity test, and exploratory factor analysis to verify construct validity (see Table 2).

#### 3.4.3. Wellness Questionnaire

This study edited and revised the wellness questionnaire developed by Russell [41]. The questionnaire consists of two dimensions, namely Mental Health, Physical and Mental Harmony and Physical Health. Scores are measured on a five-point Likert scale, with the measures being strongly agree (5 points), agree (4 points), neutral (3 points), disagree (2 points), and strongly disagree (1 point). The higher the score, the stronger the participants’ perception of good wellness, and vice versa. The internal consistency of the scale expressed as the Cronbach’s alpha is 0.876, suggesting that the questionnaire has good reliability. This research uses the KMO test for goodness of fit, Bartlett’s sphericity test, and exploratory factor analysis to verify construct validity (see Table 3).

### 3.5. Analysis Tools and Methods

The research applied SPSS 25.0 statistical software for data analysis. The data is analyzed in terms of frequency distribution, percentage, mean, Pearson correlation coefficient, and multiple regression.

## 4. Results

### 4.1. Description of Participants’ Personal Background

The primary characteristics of the participants are as follows: in terms of gender, there were more women (150) than men; in terms of educational background, most of the participants (220) held an undergraduate degree or above; in terms of tenure, most of them (195) had worked for 15 years in the hospitality industry; in terms of position, most of the participants (180) were supervisors; and in terms of salary, most of the participants (150) earned between US$1000 and US$2000 per month.

### 4.2. Analysis of the Current State of Organisational Climate

The total mean of organizational climate was 3.48 (standard deviation (SD) = 0.64). Concerning its dimensions, responsibility was the highest (mean = 3.7; SD = 0.72), followed by identification (mean = 3.47; SD = 0.78), and reward and promotion incentive (mean = 3.42; SD = 0.71) All dimensions were higher than the theoretical midpoint (3 points), with responsibility having the highest mean. This part measures the current state of organizational climate. Therefore, the standard deviation is small, which means that the questionnaire titles are suitable and homogeneous.

### 4.3. Analysis of the Current State of Job Stress

The total mean of job stress was 3.11 (SD = 0.47). Concerning its dimensions, interpersonal stress was the highest (mean = 3.34; SD = 0.65), followed by workload stress (mean = 3.23; SD = 0.55), and supervisor support stress (mean = 2.68; SD = 0.63). The last item is professional competence stress (mean = 2.58; SD = 0.63) Interpersonal stress had the highest mean, while supervisor support stress had the lowest mean. This part measures the current state of job stress. Therefore, the standard deviation is small, which means that the questionnaire titles are suitable and homogeneous.

### 4.4. Analysis of the Current State of Wellness

The total mean of wellness is 3.49 (SD = 0.43). Concerning its dimensions, mental health was the highest (mean = 3.59; SD = 0.50), followed by physical health (mean = 3.32; SD = 0.62), and physical and mental harmony (mean = 3.42; SD = 0.56). All dimensions were higher than the theoretical midpoint (3 points), with mental health having the highest mean. This part measures the current state of wellness. Therefore, the standard deviation is small, which means that the questionnaire titles are suitable and homogeneous.

### 4.5. Correlation Analysis of Organisational Climate, Job Stress and Wellbeing

According to the result of the Pearson correlation matrix in Table 4, most of the correlation coefficients of the dimensions of organizational climate, job stress, and wellness in the hospitality industry were at least moderate. Therefore, Hypothesis 1 is supported.

### 4.6. Multiple Regression Analysis of Personal Background Factors, Organizational Climate, Job Stress and Wellness

According to the multiple regression analysis in Table 5, gender, educational background, seniority, position, salary, reward and promotion incentive, identification, responsibility, and other variables can associated with an employee’s wellness. Therefore, Hypothesis 2 is supported.

According to the multiple analysis result in Table 6, gender, educational background, seniority, position, salary, supervisor support stress, professional competence stress, workload stress, interpersonal stress, and other variables can associated with an employee’s wellness. Therefore, Hypothesis 2 is supported.

## 5. Discussion

According to the analysis results, there is a significant and negative correlation between the perception of organizational climate and job stress in all dimensions, indicating that the higher negative perception of organizational climate, the greater the perception of job stress. There is a significant and positive correlation between the perception of organizational climate and wellness in all dimensions, suggesting that the higher the perception of organizational climate, the greater the perception of wellness. Finally, there is a significant and negative correlation between the perception of job stress and wellness in all dimensions, indicating that the greater the perception of job stress, the lower the perception of wellness. These results are in line with those from previous studies [11,27,46].

It is worth noting that the mean value of the item “As long as I think it is correct, I would go ahead with it and there is no need to ask for instructions (M = 2.86)” in the Organizational Climate Questionnaire is smaller than the median, and the supervisor support stress dimension in the job content questionnaire (M = 2.79) is also smaller than the median. In addition, the mean value of the second item in the Wellness Questionnaire, “I have no doubts about my ability and judgment at work, and I will continue to improve myself-confidence (M = 3.72)”, is higher than the median. Based on these results, hospitality employees were rather confident of their professional attitudes and job competence, but they are seldom identified or favored by others. This suggests that, despite their confidence, they do not have much authority in their work tasks. Therefore, this study suggests that hospitality business owners should not only provide employees with complete education and training to empower their professional knowledge but should also provide them with sufficient authority, to jointly maximize interests and contributions to the hospitality industry.

Cumulative job stress associated with employee wellness. We find from the analysis of job stress that the construct of “interpersonal stress” has the highest score, which means that employees feel the greatest increase in stress due to poor coordination and communication with colleagues during COVID-19. “Workload stress” is also a major contributor, in which workloads generate additional stress due to vague divisions of powers/responsibilities and insufficient time for completing tasks. Previous studies show that organizational structures in the hospitality industry need to be revised effectively, and it is very important to effectively reduce the job stress of employees [48,49]. Organizational climate and job stress are key indicators of organizational operations [45]. Therefore, in light of the changes and globalized competition during the COVID-19 pandemic, the hospitality industry should take a more positive stance in making strategic plans to change the organizational structure climate, so that employees have a better perception of the overall organizational climate [25]. Meanwhile, employees should also cooperate with the adjustment of their organization and pay more attention to their wellness. Only through good wellness management can the quality of the industry be enhanced, and companies should constantly maintain sustainability in their organizational climate.

## 6. Conclusions

According to the regression analysis results, position, identification, and reward and promotion incentives in various organizational climates, were associated with the wellness of employees, indicating that the most effective way to improve organizational climate and wellness is to provide rewarding and promotional incentives in all dimensions of the organizational climate. Therefore, it is suggested that hospitality business owners can provide an effective remuneration and reward system, enhance organization identification, provide timely encouragement, and appropriately adjust the organizational environment to enhance their employees’ perception of wellness.

The dimensions of job stress—i.e., supervisor support stress, professional competence stress, workload stress, and interpersonal stress—can obtain a correlation between physical and mental health. There was a negative correlation between supervisor support stress and professional competence stress, while workload stress and interpersonal stress had positive correlations with mental and physical health. This study concludes that supervisor support stress and professional competence stress arise from a poor understanding of job contents. When professional competence stress and supervisor support stress are lessened, this has associated effects on employee wellness. However, workload stress and interpersonal stress positively correlate with physical and mental health, demonstrating that the greater the workload stress, the poorer the perception of wellness.

## 7. Research Limitations

This research was mainly conducted on employees of five hotel branches in Taipei City, Taiwan. Therefore, the results could not be applied to the entire hospitality industry. Meanwhile, different companies have special cultural backgrounds, core values, and organisational structures, and this research can merely serve as a reference for human resource management and organisational adjustment. Further research is needed concerning the organisational structure, cultural background, core values, and regional characteristics of other industries.

## 8. Practical and Managerial Implications

The topic of this study is the impact of organizational climate and job stress on the well-being of hospitality employees. This study belongs to the category of environmental psychology. In the past, most studies on organizational climate focused on innovative performance, organizational innovation, organizational leadership, work attitudes, and turnover intentions. There is a dearth of studies on the impact of organizational climate and job stress on hospitality employees’ wellness in Taiwan during the COVID-19 pandemic. By centering on organizational climate, job stress, and wellness, this study sheds further light on how hospitality business owners create a suitable organizational climate and effectively reduce the job stress of employees during the COVID-19 pandemic, to improve their wellness and lead the way towards sustainability. As the research shows, the three variables are interrelated, so employers should try to improve organizational climate. Additionally, reducing job stress and safeguarding wellness during COVID-19 should be a priority. We recommend that hotel operators adopt measures to improve organizational climate, mitigate job stress, and maximize the wellness of their employees.

## Figures and Tables

**Table 1:** Summary table of factor analysis of organizational climate scale.

Number	Question Title	Factor 1	Factor 2	Factor 3
		Reward and Promotion Incentive	Identification	Responsibility
1	During COVID-19, the company is often encouraging and supportive of personal judgements, as long as it helps the supervisor.	0.833		
2	During COVID-19, the rewards and encouragement that employees receive are more than accusations and criticisms.	0.786		
3	During COVID-19, the company rewards and praises good work performance.	0.781		
4	During COVID-19, production is positively affected by good planning.	0.777		
5	During COVID-19, employee rewards are proportional to work performance.	0.754		
6	During COVID-19, the company encourages employees to seek promotion.	0.767		
7	During COVID-19, employees are encouraged to seek solutions when they make mistakes.	0.729		
8	During COVID-19, if I think it is correct, I will go ahead with it and there is no need to ask for instructions	0.689		
9	During COVID-19, I will contact co-workers and be supportive of the company’s policies.		0.905	
10	During COVID-19, I am supportive and approve of new company regulations.		0.775	
11	During COVID-19, the company encourages us to be frank about our opinions, even if they differ from the supervisor’s.		0.759	
12	During COVID-19, the duties of the supervisor are to set specific working goals and help achieve them.			0.856
13	During COVID-19, the company’s policies and employee duties are clear.			0.721
Characteristic value		6.127	1.531	1.246
Explained variation (%)		47.115	11.769	9.582
Cumulative explained variance (%)		47.135	58.892	68.484
KMO Measure of Sampling Adequacy = 0.792				
Cronbach’s Alpha value = 0.896				

**Table 2:** Summary table of factor analysis in job content scale.

Number	Question Titles	Factor 1	Factor 2	Factor 3	Factor 4
		Workload Stress	Interpersonal Stress	Supervisor Support Stress	Professional Competence Stress
1	For the impact of COVID-19, I feel that workloads are currently too heavy.	0.884			
2	For the impact of COVID-19, the unclear division of powers and responsibilities in the company doubles my work and halves the results.	0.849			
3	For the impact of COVID-19, I have too much work to complete in the allotted time.	0.847			
4	For the impact of COVID-19, I feel a lot of pressure to reach company goals.	0.825			
5	For the impact of COVID-19, I feel a lot of pressure every time my performance fails to meet company goals.	0.798			
6	For the impact of COVID-19, my job is often hard to complete alone. Colleagues may need to help one another.	0.790			
7	For the impact of COVID-19, I am troubled by insufficient coordination among colleagues		0.745		
8	For the impact of COVID-19, I often remain silent to maintain the peace between colleagues.		0.743		
9	For the impact of COVID-19, I worry about conflicts with colleagues due to miscommunication and poor coordination.		0.741		
10	For the impact of COVID-19, I feel work is busy, which makes it hard to maintain positive relationships with my colleagues.		0.707		
11	For the impact of COVID-19, I feel my supervisor believes I lack the required ability.			0.864	
12	For the impact of COVID-19, I feel the supervisor ignores or demeans my work performance.			0.853	
13	For the impact of COVID-19, I am unable to finish all of the tasks required by the supervisor.			0.836	
14	For the impact of COVID-19, I feel little sense of achievement in the job.			0.709	
15	During COVID-19, I feel I’m not always sufficiently professional.				0.855
16	For the impact of COVID-19, I am unable to show my competence.				0.798
17	For the impact of COVID-19, I am unable to show my professional competence in my job.				0.769
18	For the impact of COVID-19, I cannot convince my co-workers of what to do.				0.740
19	I feel my professional development is slower. than the speed of the spread of COVID-19.				0.729
20	For the impact of COVID-19, I feel helpless about finding the time for professional development.				0.665
Characteristic value		5.876	2.792	1.606	1.235
Explained variation (%)		39.171	18.612	10.705	9.682
Cumulative explained variance (%)		39.171	57.783	68.488	78.170
KMO Measure for sampling adequacy = 0.751					
Cronbach’s Alpha value = 0.876					

**Table 3:** Summary table of factor analysis for wellness scale.

Number	Question Titles	Factor 1	Factor 2	Factor 3
		Mental Health	Physical and Mental Harmony	Physical Health
1	During COVID-19, I am full of energy for facing work challenges.	0.805		
2	During COVID-19, I have no doubts about my ability and judgment at work, and I will continue to improve myself.	0.804		
3	During COVID-19, I won’t feel sad or depressed when encountering frustrations.	0.790		
4	During COVID-19, I seldom feel overwhelmed or worried.	0.777		
5	During COVID-19, if my colleagues and friends shun me, I will examine what I have done and try to maintain good relationships.	0.749		
6	During COVID-19, I won’t stay in bed for job burnout.	0.727		
7	During COVID-19, I am seldom exhausted by heavy burdens.		0.850	
8	During COVID-19 in my usual work, I am often busy but rarely upset or feel ill at ease.		0.834	
9	During COVID-19, when stress from my job starts to rise, I have enough energy to overcome most challenges.		0.787	
10	During COVID-19, when I feel angry at work, I often remind myself that “It’s good to be alive”, which helps to adjust my emotions and lessen the stress.		0.515	
11	During COVID-19, I am healthy and free of most aches and pains (such as in my head, arms, shoulder, waist, and feet).			0.798
12	During COVID-19, I never feel short of breath or dizzy.			0.769
13	During COVID-19, I feel no muscle tremors (such as eyelid twitches).			0.740
14	During COVID-19, my weight is normal; it hasn’t changed.			0.665
Characteristic value		5.529	2.180	1.591
Explained variation (%)		39.495	15.568	11.366
Cumulative explained variance (%)		39.495	55.063	66.429
KMO for sampling adequacy = 0.751				
Cronbach’s Alpha value = 0.876				

**Table 4:** Correlation analysis of wellness, organizational climate, and job stress in the hospitality industry.

	Reward and Promotion Incentive	Identification	Responsibility	Supervisor Support Stress	Professional Competence Stress	Workload Stress	Interpersonal Stress	Mental Health	Physical and Mental Harmony	Physical Health
Reward and promotion incentive	1									
Identification	0.71 **	1								
Responsibility	0.46 **	0.55 **	1							
Supervisor support stress	0.08	0.03	0.05	1						
Professional competence stress	0.09	0.04	0.05	0.08	1					
Workload stress	−0.17 **	−0.12 *	−0.09	0.42 **	0.41 **	1				
Interpersonal stress	−0.25 **	−0.32 **	−0.15 **	0.38 **	0.35 **	0.48 **	1			
Mental health	0.33 **	0.35 **	0.19 **	0.10	0.12	0.00	−0.11	1		
Physical and mental harmony	0.33 **	0.31 **	0.22 **	0.15 **	0.15 **	−0.09	−0.10	0.71 **	1	
Physical health	0.18 **	0.21 **	0.07	−0.05	−0.06	−0.19 **	−0.17 **	0.33 **	0.48 **	1

Note: * *p* < 0.05, ** *p* < 0.01.

**Table 5:** Multiple regression analysis of wellness, personal background factors, and organizational climate.

Personal Background	Mental Health		Physical and Mental Harmony		Physical Health	
	Beta	t	Beta	t	Beta	t
Gender	−0.07	−0.62	−0.03	−0.86	0.01	0.21
Educational background	0.04	0.82	0.04	0.77	0.11	1.75
Seniority	0.12	1.36	−0.03	−0.18	−0.02	−0.12
Position	−0.12	−2.10 *	−0.03	−0.56	0.06	1.16
Salary	0.02	0.18	0.03	0.33	0.06	0.84
Reward and promotion incentive	0.16	1.84	0.23	3.12 *	0.11	1.53
Identification	0.28	3.38 *	0.14	1.57	0.13	1.62
Responsibility	−0.07	−1.20	0.01	0.17	−0.03	−0.64
Constant	2.85		2.46		2.36	
R*^2^*	0.16		0.13		0.07	
F	7.98 ***		5.95 ***		2.92 *	

Note: *p < 0.05, ***p < 0.001.

**Table 6:** Multiple regression analysis of wellness, personal background factors, and job stress.

Personal Background	Mental Health		Physical and Mental Harmony		Physical Health	
	Beta	t	Beta	t	Beta	t
Gender	−0.03	−0.55	−0.06	−1.06	0.02	0.16
Educational background	0.02	0.24	0.02	0.33	0.08	1.38
Seniority	0.06	0.78	0.00	0.05	−0.04	−0.42
Position	−0.12	−1.88	−0.03	−0.36	0.04	0.58
Salary	0.13	1.51	0.11	1.17	0.13	1.63
Supervisor support stress	0.14	2.28 *	0.26	3.85 ***	0.07	1.29
Professional competence stress	0.11	2.39 *	0.28	3.38 ***	0.06	1.38
Workload stress	0.00	0.06	−0.15	−2.09 *	−0.14	−2.22 *
Interpersonal stress	−0.18	−2.95 **	−0.15	−1.92	−0.11	−2.03 *
Constant	3.68		3.57		3.73	
R*^2^*	0.06		0.07		0.07	
F	3.53 *		3.18 **		2.83 **	

Note: *p < 0.05, **p < 0.01, ***p < 0.001.

## Data Availability

Not applicable.
